# Miasm and Malaria

**Published:** 1876-05

**Authors:** 


### MIASM AND MALARIA

Are the great death agents throughout the largest portion of the habitable globe.

Miasm is Malaria, but Malaria is not Miasm.

Miasm is an emanation from decaying vegetation. Malaria is bad air, whatever may be its source. All impure air is Malaria.

Miasm is so rarified by a sun of ninety degrees, that it rises rapidly above us, and is innocuous. The cool of the morning and evening of summer time condenses it, and causes it to fall to the surface of the earth, where it is breathed by man, and is the fruitful cause of pestilence, plague, and epidemic fevers. Thus the higher persons sleep above the surface of the earth, the healthier is the atmosphere.

While as a general rule it is better to sleep in apartments having a window and the fireplace open in all seasons, yet, where miasm abounds, evidencing its presence by chills and fever, fever and ague, diarrhœas, and the like, it is better to sleep with closed windows than to have them open, because men are known to fatten in jails and small prison cells, while the breathing of malaria a single night has originated diseases

